# Supplementary material for: Inflammatory factors and risk of lung adenocarcinoma: a Mendelian randomization study mediated by blood metabolites
Source: Front Endocrinol (Lausanne). 2024 Aug 27;15:1446863. doi: 10.3389/fendo.2024.1446863 (PMC11384989; doi:10.3389/fendo.2024.1446863)
Supplement: Supplementary file 1 [file DataSheet1.zip › supplementary files/S2/GCST90200384/sensitivity-analysis.pdf]

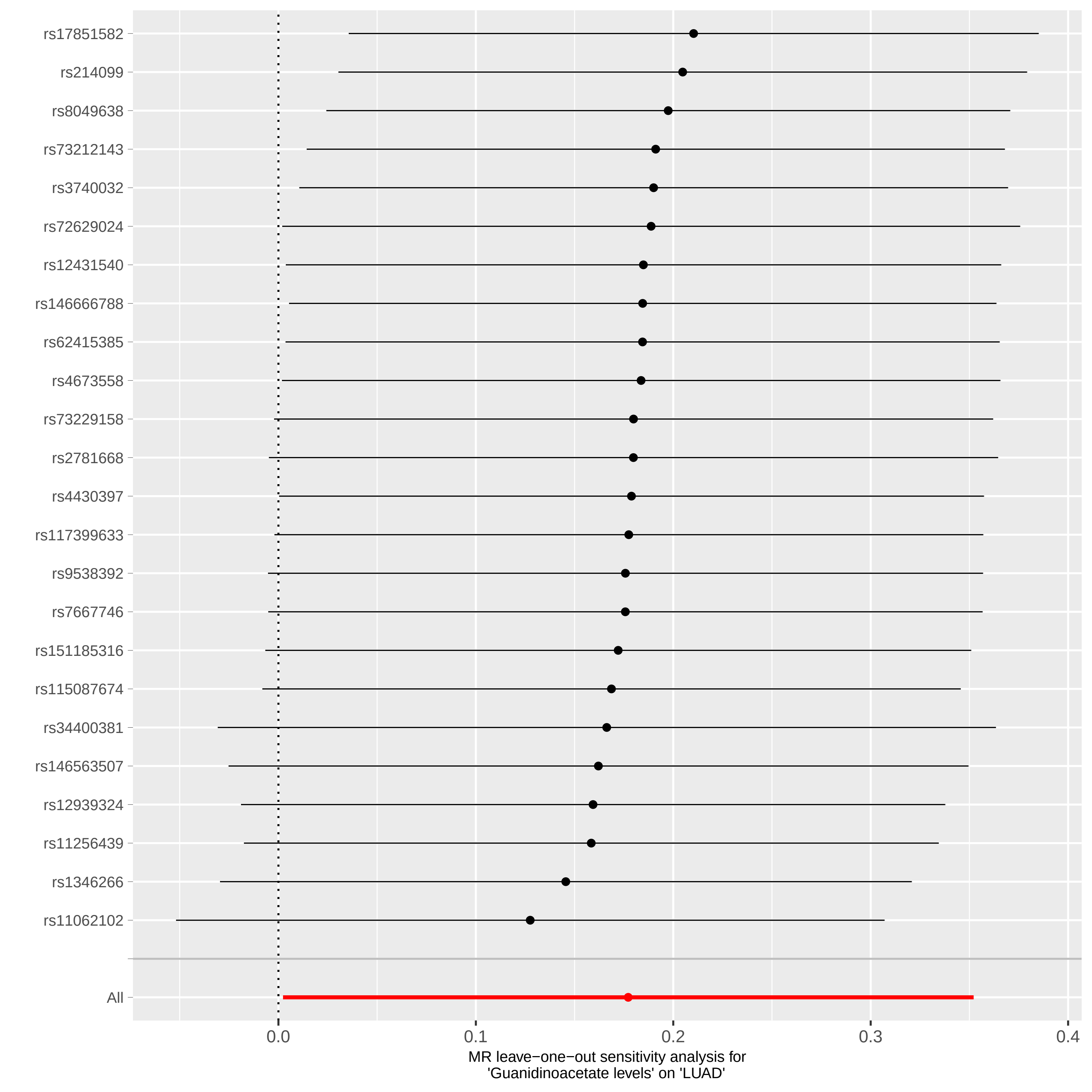
rs17851582
rs214099
rs8049638
rs73212143
rs3740032
rs72629024
rs12431540
rs146666788
rs62415385
rs4673558
rs73229158
rs2781668
rs4430397
rs117399633
rs9538392
rs7667746
rs151185316
rs115087674
rs34400381
rs146563507
rs12939324
rs11256439
rs1346266
rs11062102
All
0.0
0.1
0.2
0.3
0.4
MR leave−one−out sensitivity analysis for
'Guanidinoacetate levels' on 'LUAD'
